# Experimental study on the damage performance and forced response of concrete lining in fault-crossing tunnel

**DOI:** 10.1038/s41598-024-52318-7

**Published:** 2024-01-24

**Authors:** Hui Hu, Youdi Lan, Yun Liu, Wenge Qiu

**Affiliations:** 1 Key Laboratory of Transportation Tunnel Engineering, Ministry of Education, School of Civil Engineering, Southwest Jiaotong University, Chengdu, 610031 Sichuan China; 2 Department of Road and Bridge Engineering, Sichuan Vocational and Technical College of Communications, Chengdu, 611130 China

**Keywords:** Civil engineering, Natural hazards

## Abstract

Understanding the adverse effects of tunnel crossing active faults on tunnel structures is crucial for ensuring their safe operation and construction. This paper presents the results of a series of model tests conducted at a scale of 1:40 using a fault sliding test box. Three sets of fault comparison tests were carried out, namely: (1) the tunnel does not cross the fault, (2) the spring stiffness is reduced, and (3) the model is not reinforced. The objective was to study the failure characteristics of tunnels crossing active faults. The findings reveal that when the hanging wall moves downwards, cracks appear on the surrounding rock surface of the hanging wall, specifically above the tunnel lining crossing the fault. The lining is significantly damaged within the range of − 30–+ 30 cm. All points of axial force exhibit an increasing compression trend. The section of axial force and bending moment near the fault fracture surface is notably larger than that far from the fault fracture surface. The safety factor of the entire structure decreases sharply after dislocation, making the tunnel more susceptible to cracking at various locations such as the vault, arch waist, left and right arch feet, and inverted arch. It has been proven that the shear compression of the fracture surface during fault dislocation is the main cause of longitudinal through cracks in the lining. The use of springs with higher stiffness effectively ensures the reciprocating dislocation of the upper and foot walls, with long duration and large displacement, providing a better simulation of the dislocation of active faults.

## Introduction

**Figure 1:**
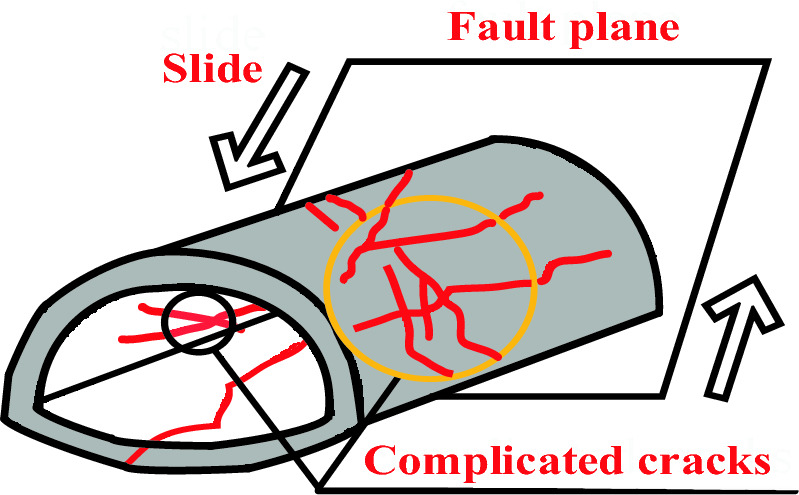
Tunnel damage pattern caused by fault slide.

A growing number of mountain tunnels will unavoidably be built on the active fault zone in southwest China in the next years due to advancements in tunnel construction technology and rising traffic demand in the country; some of these tunnels will even traverse the active fault and its effect zone. The engineering community has taken notice of the detrimental implications that the displacement of the active fault is having on the tunnel construction. To ensure the safe operation and construction of tunnels, it is therefore vital to examine the seismic mechanism of tunnels spanning active faults. Mountain tunnels are no longer thought to be more susceptible to earthquake damage than ground structures, as evidenced by a number of examples of tunnel earthquake-related damage that have been reported in recent years. The 1995 southern Hyogo earthquake and the 1999 Chi Chi earthquake in Taiwan Province both seriously destroyed numerous transportation tunnels^1,2^. Based on observations, fault displacement, slope instability, soil liquefaction, and seismic wave propagation are the main causes of catastrophic damage to mountain tunnels^3^. The tunnel traversing the active fault is particularly vulnerable to fault dislocation, which can result in lateral tunnel deformation, complicated lining cracks, and tunnel axis distortion, as illustrated in Fig. 1^4^. For instance, the Baiyunding Tunnel on the Duwen Highway (which is situated in the southwest of China) sustained extensive lining displacement, peeling, and damage during the Wenchuan earthquake (Ms = 8.0) in 2008. Numerous additional transit tunnels have also sustained damage, albeit in different amounts, as a result of the collapse brought on by fault displacement^5^. People are gradually becoming more interested in the study of tunnel structure seismic performance as the amount of earthquake damage to tunnel structures increases. Analysing how an earthquake may affect the structure of tunnel engineering is therefore a critical task for civil engineers^6^.

According to the earthquake damage investigation, it is found that the tunnel crossing the fault is the most seriously damaged^7–9^. Therefore, in recent years,a lot of research work has been carried out around the earthquake damage mechanism and anti-vibration technology of fault tunnels^10^. Shaking table test is widely used in the study of tunnel seismic mechanism under fault displacement. For example, in 1987, Yakovlevich et al. used the shaking table to study the response of tunnel lining^11^; In 1984,Shunzo Okamoto et al. carried out experiments on underwater tunnels with the help of mechanically excited vibration table^12^. In 1988, Goto Y et al. of Japan conducted a model test on the seismic response of two parallel shield tunnels, and studied the influence of parallel spacing on the seismic response of the structure^13^. In 1993, Zhiying Xu et al. made a large-scale shaking table test and calculationand studied the dynamic interaction between soil and underground structure under the condition of dynamic input^14^. In 1996, Changqing Shi et al. conducted an experimental study on the seismic performance of shallow-buried and open-cut subway station structures, and analyzed the typical seismic problems of urban subway stations under shallow-buried conditions^15^. In 2002, Qianqian Ji et al. made a shaking table model test of subway station structure^16,17^. Bining Gong completed the experimental study on the dynamic interaction between underground structure and soil^18^. Dapeng Zhao et al. conducted experimental research on the vibration behavior of long-span underground structures^19^. Lincong Zhou et al. have carried out shaking table test research on underground structure earthquake simulation^20^. In 2006, Haiyang Zhang et al. did some research on nonlinear dynamic interaction of soil-underground structure and its large-scale shaking table test, and achieved some results^21^. Lianjin Tao et al. carried out shaking table model tests on typical subway station structures^22^. Ailan Che et al. completed the model vibration test and numerical analysis of subway seismic response^23^. Kailing Li et al. conducted a model test analysis of soil-subway tunnel dynamic interaction^24^. In 2007, Guoxing Chen et al. made a large-scale shaking table model test study on soil-subway station structure dynamic interaction^25^. Xiaojun Shi et al. have completed the large-scale shaking table model test of underground utility tunnel^26^. In 2012, Guangyao Cui made an anti-seismic test of the stick–slip section of the fracture, and studied the influence law and scope of fault dislocation on the tunnel, the optimal spacing of damping joints, the optimal thickness of damping layer and the damping effect of damping joints on the initial support, and achieved some results^27^. Y.S. Shen et al. to improve the seismic performance of the mountain tunnel through fault, a design idea or method of the between sectional tunnel structures with the flexible joint were put forward to run through the active fault and verified or analyzed by using the shaking table test^28^. Sujian Ma et al. relying on an actual tunnel in the southwest mountainous area to establish a three-dimensional finite element model, the failure mechanism of the tunnel under strike-slip and thrust fault dislocation is revealed from the lining deformation, stress distribution, and plastic zone distribution, and the results show that the damage range of the lining distributes in the area of the fracture and the damage effect is greatly affected by the movement amount of the active fault^29^. Milad Zaheri et al. used three-dimensional numerical simulation to study the effects of strike-slip fault movement on the performance of shotcrete and segmental linings in shallow tunnels that transversely cross the fault^30^. Q.P. Cai et al. has studied through centrifugal model test and numerical analysis to investigate the deformation mechanisms of an existing tunnel due to normal faulting in sand^31^. Wang Zhen et al. studied the structural response and failure mechanism of water conveyance tunnel under the action of reverse fault^32^. Cui Zhen et al. studied the response and mechanism of a tunnel subjected to strike-slip fault rupture through model test and DEM-FDM coupling numerical analysis. The interaction of the tunnel with the fault rupture, the deformation pattern, and the strain evolution and crack propagation in the tunnel liner were observed in the test^33^. Masoud Ranjbarnia et al. studied the influence of reverse fault and normal fault movement on lateral crossing of shallow shotcrete tunnel^34^. Zaheri Milad et al. investigated the effects of a dip-slip fault (a normal or a reverse fault) movement on a segmental tunnel which transversely crosses either of this kind of faults^35^. Majid Kiani et al. described nine centrifuge modeling details of normal fault and segmented tunnel, and investigated the influence of overburden and fault angle change on tunnel behavior^36^. Aghamolaei Milad et al. recorded and discussed the axial force, bending moment and rotation angle when the tunnel suffered from reverse fault dislocation under different vertical fault offset^37^. Ghadimi Chermahini et al. used explicit dynamic analysis method to consider the influence of fault movement. Different influencing factors, such as tunnel location, intersection angle, inclination angle and soil characteristics around the tunnel, are studied^38^. Majid Kiani et al. proposed an experimental method to create the brittle curve of shallow tunnels in alluvium affected by normal surface faults^39^. Mehdi Sabagh et al. carried out a series of centrifugal model tests for shallow tunnels crossing normal faults; Shows the observed ground deformation, fault scarps and sinkholes caused by tunnel damage^40^. Mohammad Hazeghian et al. uses DEM modeling method with rolling resistance based on GPU to comprehensively study dip-slip faults passing through granular soil from engineering and foundation perspectives^41^. Most of these experimental studies focus on the simulation of the seismic performance and damping methods of urban subways, especially subway stations. However, there are few studies on the outstanding seismic response of mountain tunnels, especially the failure characteristics of long tunnels passing through active faults, so this specific research field has not been well understood.

In this paper, a self-designed fault dislocation test box is used to carry out similar model failure characteristics tests of tunnels crossing active faults, as well as comparative tests under different conditions, which reproduce the mechanical behavior characteristics of tunnels crossing active faults, and analyze the failure of surrounding rock and lining structure after tunnel tests, vibration wave analysis caused by dislocation and internal force analysis of secondary lining, establish a numerical simulation test, and compare and analyze the differences between the results of numerical simulation and model test. The research results can provide scientific guidance and reference value for similar tunnel engineering seismic response problems.

## Construction of simulation test platform

### Test device system

**Figure 2:**
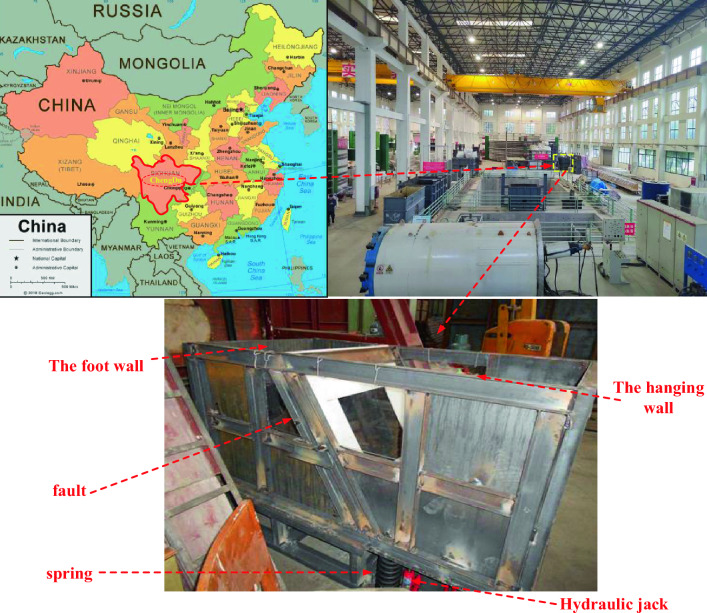
Overview of fault dislocation test box. Note: The map in figure is from https://zhfw.tianditu.gov.cn/.

The fault dislocation test box, several sets of springs and jacks, a PVC plastic plate, a tunnel lining model, a strain gauge, and a static strain collector make up the majority of the model test equipment. An original fault sliding test box was created (see Fig. 1) to comprehend the mechanism by which fault displacement influences tunnel seismic measures. It replicates the effect of fault movement on the actual tunnel lining. The test chamber is 2.0 m length, 1.0 m wide, and 1.0 m high, with no cover (as illustrated in Fig. 2). The two sections of the test room represent the foot and top walls of inclined faults, respectively. U-shaped steel and steel plates are used to weld both. Together, these two sections provide a 60-degree inclination, and the hanging wall's ability to move replicates the fault plane. To ensure smooth sliding, lubricate the inclined plane with the appropriate amount. Furthermore, two plexiglass observation windows are positioned close to the fault's dip angle so that observers may see how the simulated ground moves on each side of the fault plane when the fault slides.

**Figure 3:**
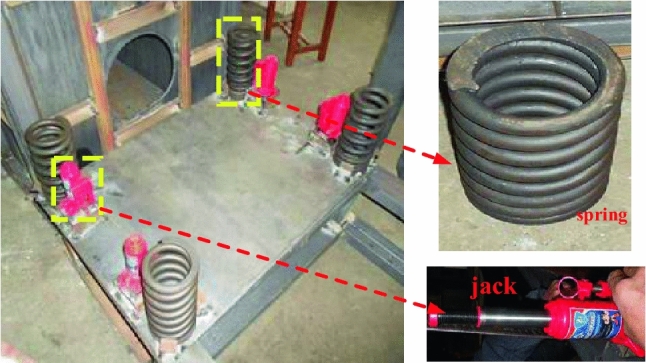
Spring and jack.

It is known that the relative motion of the hanging wall and the foot wall is a reciprocating process in the typical fault sliding process. In the experiment, the upper plate is lifted to the same horizontal position as the lower plate by the jack, and springs are placed at the four corners of the bottom of the upper plate box by using the gap formed by the jack. As shown in Fig. 3, the stiffness of the springs can be calculated to generate a compression of 3–4 cm after dislocation to simulate the final dislocation displacement. In this process, the hanging wall will reciprocate up and down under the action of the spring and interact with the foot wall to simulate the fault dislocation process.

**Figure 4:**
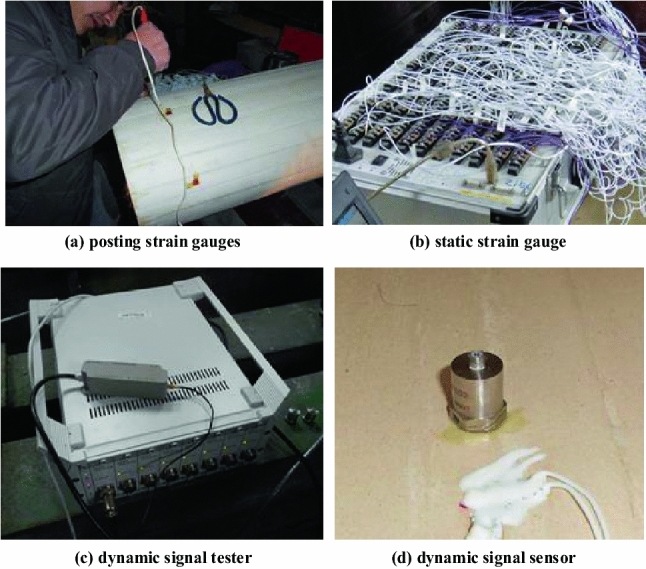
Static strain and dynamic signal acquisition.

The internal force of tunnel lining is mainly carried out by static strain gauges as shown in (a) and (b) in Fig. 4. After the lining model is cast, transverse and longitudinal strain gauges are posted at the test design position, and the distribution of wires is coordinated to ensure the intact rate of strain gauges during the test. The static strain gauge is used as shown in the figure. The vibration wave generated by fault dislocation is monitored by dynamic signal sensor as shown in (c) and (d) in Fig. 3. The instrument adopts DH3816 dynamic signal instrument, and the sensor probe is set at the inverted arch where the tunnel passes through the fault, and the sensor probe is set at the surrounding rock near the tunnel outside the fault, and the data are collected for comparative analysis.

### Similarity relation and lining model making

(1) Similarity relation

The artificial mass model is adopted, and geometry, density and cohesion are selected as basic dimensions. According to Saint–Venant principle, in order to eliminate the influence of boundary effect on test results, the distance from the model boundary to the tunnel centerline should meet at least 3–5 times the hole diameter in shaking table test. Considering the factors such as the size of model box, the section size of tunnel lining prototype and model manufacturing technology, as shown in Table 1, the geometric similarity ratio is 1: 40 and the Young's modulus similarity ratio is 1: 60. Density similarity ratio is 1: 1.5, and other physical parameters are calculated as follows: cohesion similarity ratio is 1: 40, friction angle similarity ratio is 1: 1, axial force similarity ratio is 1: 9.6 ${\times 10}^{4}$, and bending moment similarity ratio is 1: 3.84 ${\times 10}^{6}$.

**Table1:** Similarity ratio of the parameters in the test.

Name	Geometry similarity ratio/^a^	Young’s modulus similarity ratio E (MPa)/	Density similarity ratioρ (g/cm^3^)/	Cohesion similarity ratio C (Mpa)/	Friction angle similarity ratio (°)/	Axial force similarity ratio (N)/	Moment similarity ratio (N M)/
Similarity ratio	40	60	1.5	40	1.0	9.6e4	3.84 e6

^a^Physical variables with subscript refer to the prototype, and physical variables with subscript *m* refer to the model.

(2) Preparation of similar materials for tunnel lining

**Figure 5:**
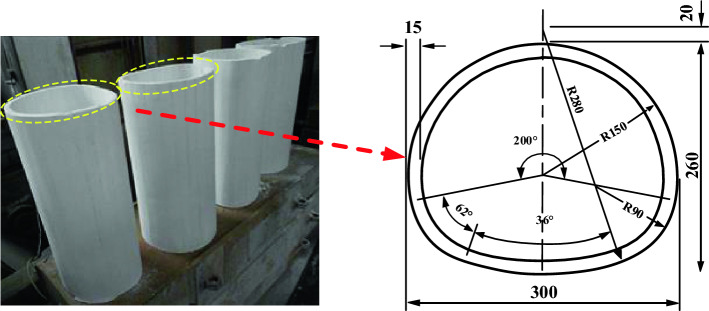
Lining section size.

The material parameters of the tunnel prototype adopt real engineering data, and the prototype and model proportions of tunnel lining parameters are shown in Table 2. The lining model is made of gypsum mixed with water (water: gypsum = 2: 3, calculated by mass percentage), and the geometric similarity ratio is 1: 40. In terms of materials, C25–C35 is used in the secondary lining of tunnel in practical engineering, and the elastic modulus is about 20–30 Pa. The secondary lining model used in the test is gypsum, and the elastic modulus is 300–500 MPa according to the material test. The tunnel is 60 cm in the longitudinal direction, and then the total length is 180 cm with gypsum after segmental pouring. The cross-sectional dimensions of tunnel lining are shown in Fig. 5.

**Table 2:** Tunnel lining parameters of the prototype and model.

Name	Poisson’s ratio μ	Young’s modulus E (MPa)	Density ρ (g/cm^3^)	Compression strength σ (MPa)
Lining prototype	0.2	3.0e4	2.6	12.2
Lining model	0.2	500	1.7	4.7

**Figure 6:**
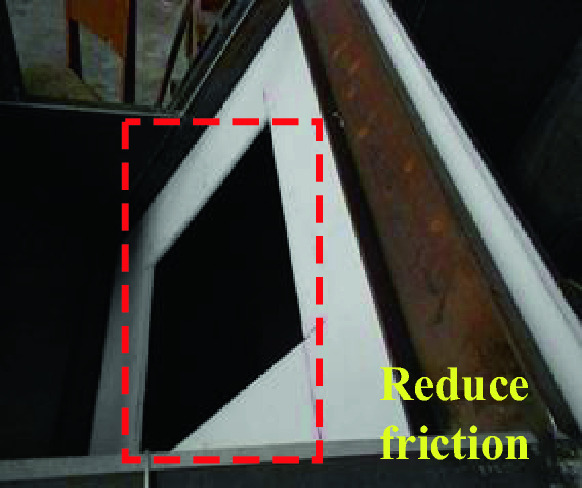
PVC plastic board.

Homogeneous materials are used to simulate the actual surrounding rock, and the parameters of prototype and model surrounding rock are shown in Table 3. Because of the complexity of the simulation test, the stratum characteristics of the actual surrounding rock are not considered. After the pre-test test, it is difficult to achieve a good dislocation effect after the upper plate is released without special treatment of friction resistance on the fault plane. After many tests, it is decided to set two PVC plastic plates with the same size on the fault plane as shown in Fig. 6 to reduce the friction resistance on the fault plane. In order not to affect the mechanical characteristics of tunnel structure near the fault, a square groove is opened in the middle of PVC plastic plate, and the groove body is far away from the lining enough to ensure that the lining is wrapped by enough surrounding rock to achieve better simulation effect.

**Table3:** Surrounding rock parameters of the prototype and model.

Name	Cohesion c (kPa)	Friction angle (º)	Young’s modulus E (MPa)	Density ρ (g/cm^3^)
Surrounding rock prototype	105.4	27.9	1.8e3	2.1
Surrounding rock model^a^	2.6	28.0	30	1.4

^a^“Surrounding rock model” equals to “Simulated ground” in the text.

### Test scheme and steps

(1) Test conditions

**Figure 7:**
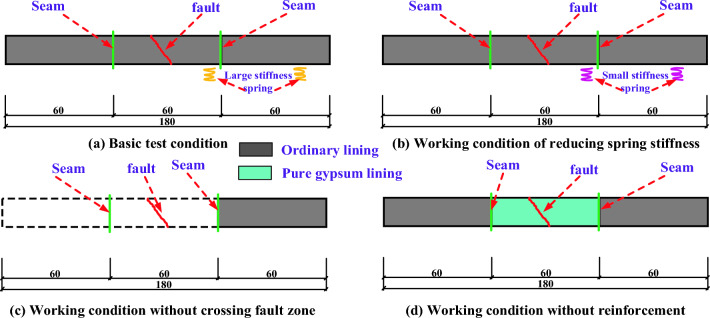
Section layout of working conditions.

The basic test condition is to simulate the failure characteristics of typical two-lane expressway tunnel and 250 km/h high-speed railway tunnel cross-section lining model when crossing active fault under the condition of Class IV surrounding rock. In order to establish a reliable test simulation method of tunnel crossing active fault model, the lining is set up without crossing fault zone, and the failure effect of fault dislocation on lining structure is compared and analyzed. Reduce the spring stiffness, and compare and analyze the destructive effect of spring reaction on lining structure; The lining is made of pure gypsum, without reinforcement. This paper analyzes the damage of plain concrete when it passes through active faults. The schematic diagram of each test condition is shown in Fig. 7, and the internal force and dynamic response of the lining are measured and analyzed.

(2) Layout of measuring points

**Figure 8:**
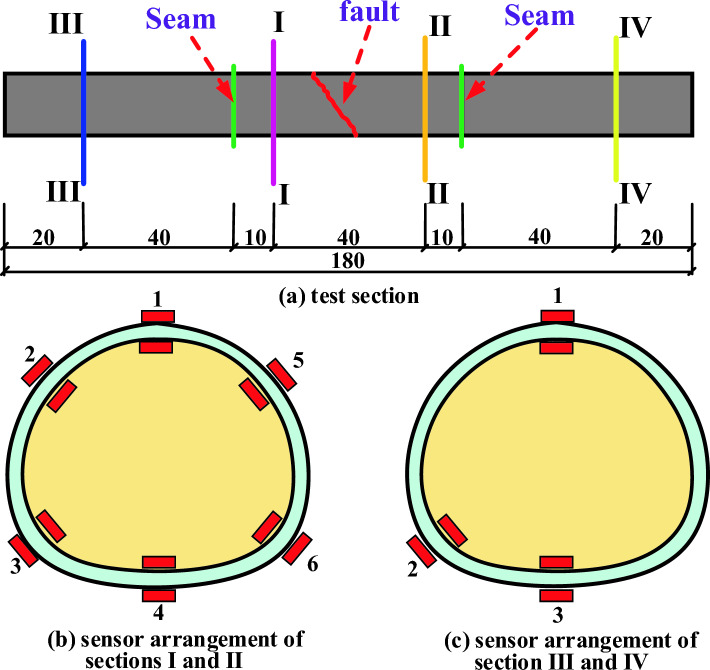
Layout of test section.

There are four sections along the longitudinal direction of the tunnel, of which I and II sections are close to the fault fracture surface, which are the key positions for testing. Each section is provided with transverse and longitudinal strain gauges inside and outside the vault, left and right arches, left and right arches and inverted arches, and the layout of test sections under various working conditions is set inside and outside the vault, arch foot and inverted arch of III and IV sections far from the fault fracture surface, as shown in Fig. 8.

(3) Test steps

**Figure 9:**
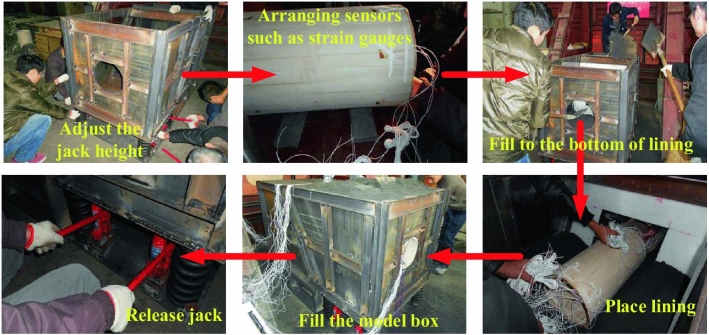
Test steps.

As shown in the Fig. 9, it is a concrete test step to simulate the failure characteristics of tunnel lining when the fault is dislocated without any anti-vibration measures. Four jacks are installed at the bottom of the upper plate part of the model box, and the stroke of the jacks is adjusted at the same time so that the upper plate and the lower plate are on a horizontal line, four springs are installed in the space formed after the jacks are pushed, static strain gauges and acceleration sensors are arranged at the key parts of the lining model, and the wires are treated as a whole, and similar materials of surrounding rock are started to be filled. Until the bottom of the preset lining position, the lining is placed, and the longitudinal joints are completed, so that the whole lining structure becomes a whole. The whole model box is filled with similar materials of surrounding rock, and compacted. During this period, pay attention to the protection of sensor data lines. After the filling is completed, the initial data of sensors are collected. At the same time, four jacks are put down, and the upper plate moves downwards and contacts the spring to generate vibration. Finally, it is connected with a computer display and the Internet to collect acceleration sensor data and lining strain data after dislocation.

## Test results

### Damage of surrounding rock and lining structure after test

**Figure 10:**
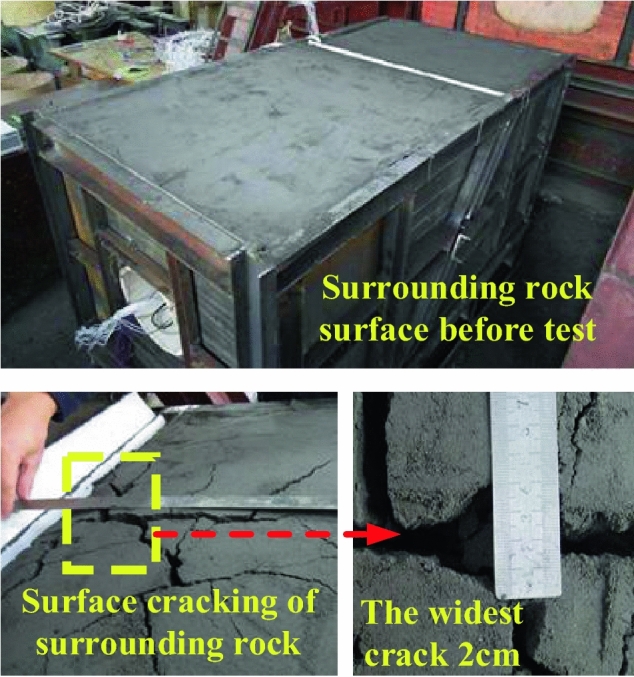
Surface condition of surrounding rock of hanging wall after test.

After the fault is dislocated, as shown in the Fig. 10, the surface of surrounding rock is compared before and after the test, and the hanging wall quickly slides down along the fracture surface and vibrates when it contacts the spring. After the vibration, the following changes have taken place in the model: there is an extended crack near the fault at the top of the surrounding rock of the hanging wall, with the widest point of 2 cm and the depth of 8 cm, and the crack area is directly above the tunnel lining crossing the fault.

**Figure 11:**
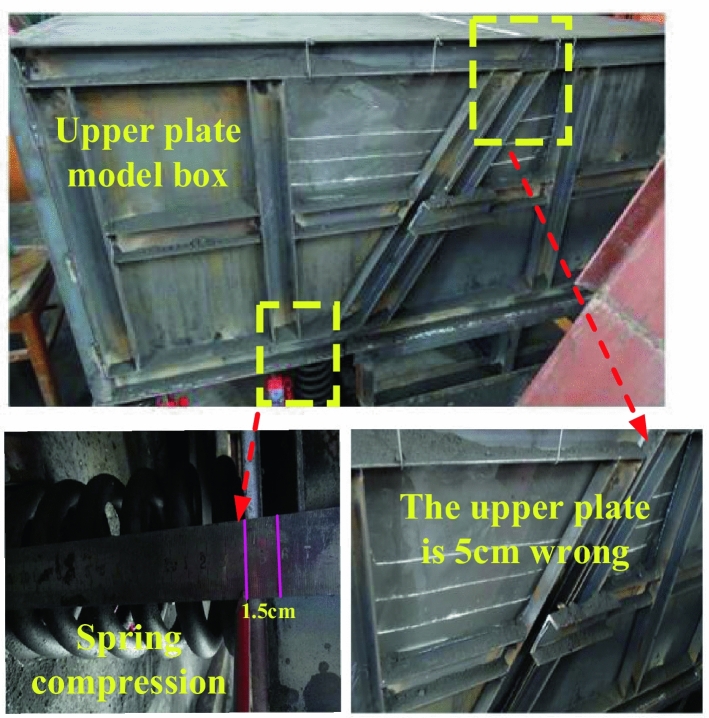
Spring compression and dislocation of upper plate model box.

The upper plate continued to oscillate until it was stable, and finally the upper plate was dislocated by about 5 cm, in which the spring was compressed by 1.5 cm. Before the two sets, the horizontal consistency changed to the top set and the bottom set, as shown in the Fig. 11.

**Figure 12:**
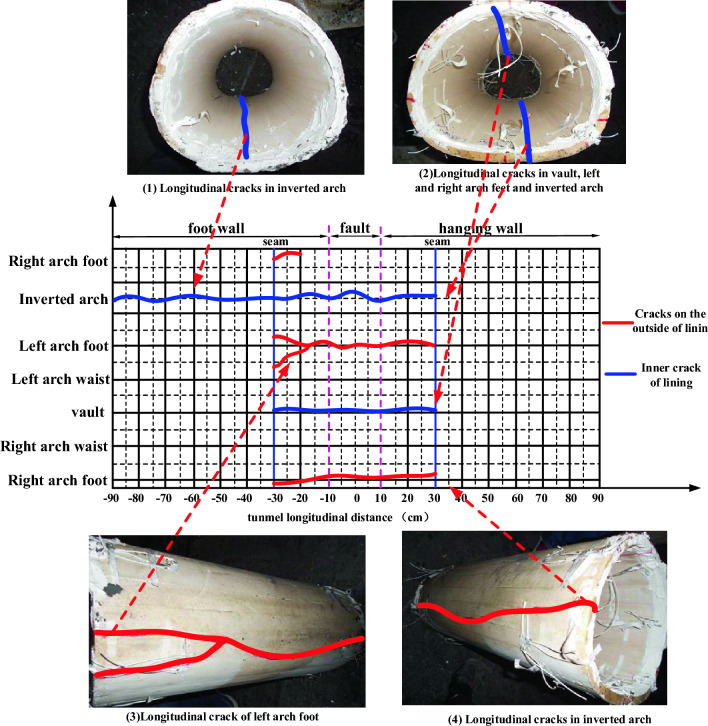
Development diagram of structural lining cracks under basic working conditions.

There are longitudinal cracks in the second lining vault, left and right arch feet and inverted arch in the fault zone, in which the vault and inverted arch are cracked and penetrated inside, and the left and right arch feet are cracked and penetrated outside; There are longitudinal cracks in the inner side of the second lining inverted arch of the outer footwall of the fault zone, and there are no cracks in other positions. Figure 12 shows the crack tracing of the lining section developed along the circumferential direction. From the Fig. 12, it can be seen that the lining is seriously damaged within the range of − 30–+ 30 cm centered on the fault fracture surface: through cracks appear in the inverted arch, and longitudinal through cracks appear in the left arch foot, right arch foot and vault. Because − 30 and 30 cm are just tunnel joints, they play a part in damping when the fault moves, and the strength of the joints is lower than that of the lining section, resulting in dislocation. In addition, there are longitudinal through cracks in the lining inverted arch in the range of −90 to − 30 cm in the footwall, which shows that the fault has a large influence on the internal force of the tunnel.

### Analysis of vibration wave caused by mismovement

**Figure 13:**
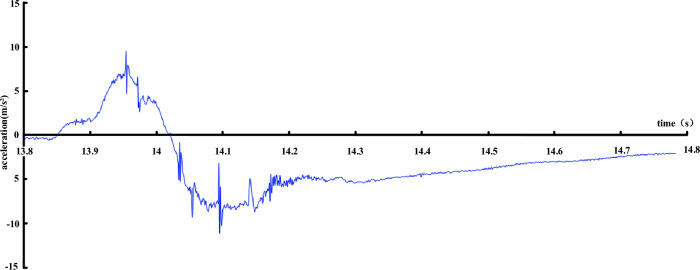
Acceleration record of surrounding rock near fault.

**Figure 14:**
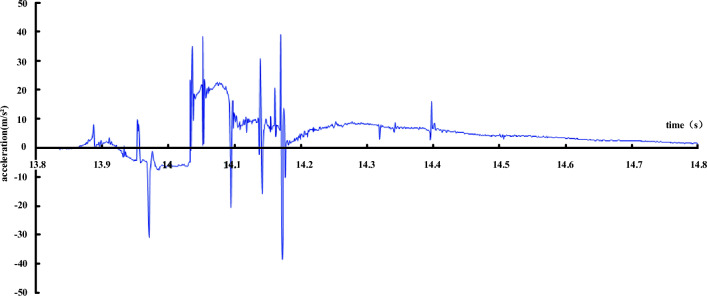
Acceleration record of lining near fault.

Figure 13 is a dynamic signal acceleration record set in the surrounding rock at the fault, and Fig. 14 is a dynamic signal acceleration record set on the lining inverted arch at the fault. As can be seen from Fig. 13, the surrounding rock and the box body are in an approximate free-fall state at the moment when the hanging wall is released, and the maximum acceleration is close to 1 g. After contacting the spring, the acceleration is reversed and continues to oscillate to stability due to the influence of the spring reaction. As can be seen from Fig. 14, the situation of lining is quite different from that of surrounding rock. Because the instantaneous acceleration of lining cracking is very large, the peak acceleration is obviously greater than that of gravity, and several large peaks appear, indicating that the lining has cracked in many places to varying degrees, and finally the acceleration continues to oscillate to stability.

### Internal force analysis of secondary lining

**Figure 15:**
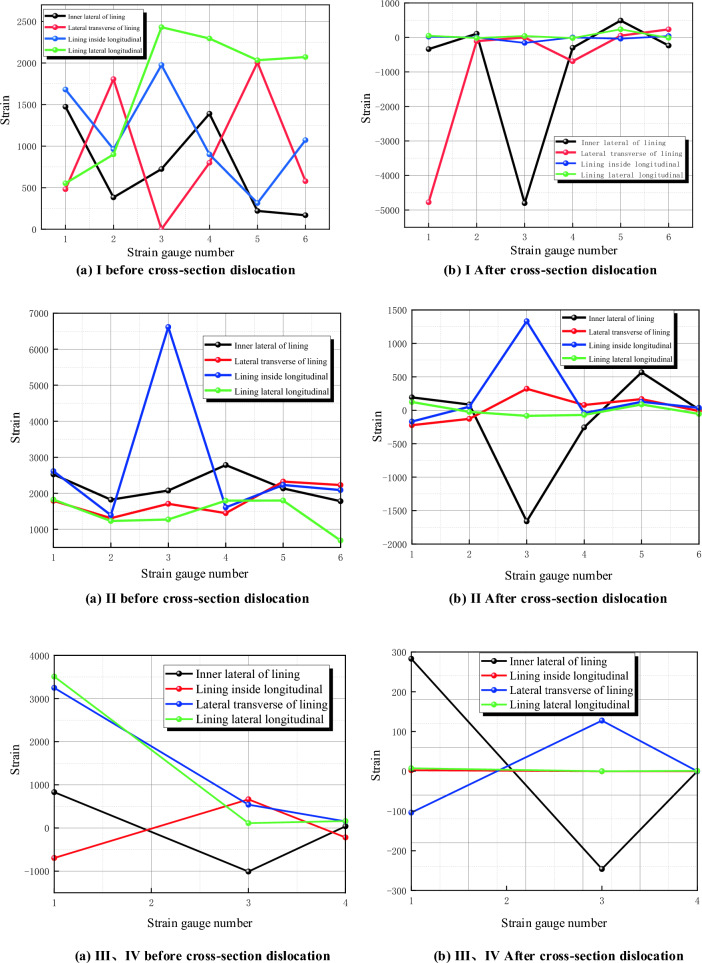
Change diagram of secondary lining before and after dislocation.

See Fig. 15 for the original data before and after the dislocation of each section of the secondary lining, and process the strain data to obtain the internal force changes of the lining structure.

#### Internal force of section

Data changes of lateral strain gauges of lining before and after fault dislocation of static strain gauge are collected and converted into axial force and bending moment, as shown in Table 4.

**Table4:** Comparison of lateral internal force of lining before and after fault dislocation without damping measures.

Section	Key position	Before and after dislocation Axial force difference (N)	Before and after dislocation Bending moment difference (Nm)	Safety margin Decreasing value
I	Vault	− 198.8016	0.4844	138.4875
	Left arch waist	− 61.5234	0.2308	69.2365
	Left arch foot	− 133.9375	− 0.3133	83.2540
	Inverted arch	− 89.3953	− 0.0282	1.1963
	Right arch waist	− 138.4250	0.3208	81.9856
	Right arch foot	− 146.8750	0.2809	62.3654
II	Vault	− 122.2594	0.4536	158.1258
	Left arch waist	− 89.2828	− 0.4198	203.9725
	Left arch foot	− 144.0844	− 0.3303	76.3255
	Inverted arch	− 124.0594	− 0.2350	53.2646
	Right arch waist	− 112.7250	0.3609	79.1564
	Right arch foot	− 124.3375	0.2809	43.2217
III	Vault	1.2656	− 0.0602	12.1569
	Arch foot	6.4266	0.0827	18.2415
	Inverted arch	5.0625	− 0.0363	10.5696
IV	Vault	7.0031	− 0.0817	13.2987
	Arch foot	4.6813	0.0037	0.0135
	Inverted arch	8.4375	− 0.0363	6.3254

From the data in the table, it can be seen that the stress of the lining structure has changed significantly before and after the dislocation of the fracture surface. The difference of internal forces before and after the dislocation can better reflect the change:

(1) Axial force

**Figure 16:**
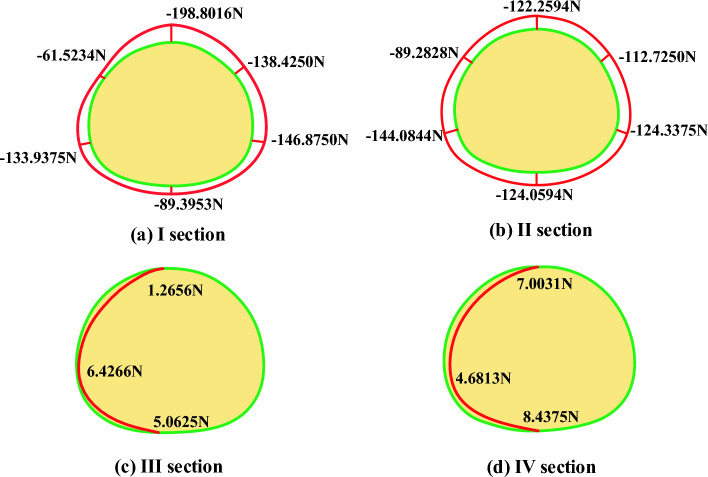
Increment of axial force after dislocation of fracture surface.

The axial force near the fault generally increases, and the changes of axial force of each section are shown in the Fig. 16: from the axial force point of view, all points show the characteristics of increasing compression, and the sections near the fault fracture surface (section I and section II) are obviously larger than those far away from the fault fracture surface, in which the maximum axial force compression increment is 198.8016 N, which occurs at the vault of section I (footwall), and the whole tunnel is squeezed circumferentially at the fault position, far from the fault. From the comparison of the upper and lower plates, the axial force increment of the two sections is close after the dislocation occurs.

(2) Bending moment

**Figure 17:**
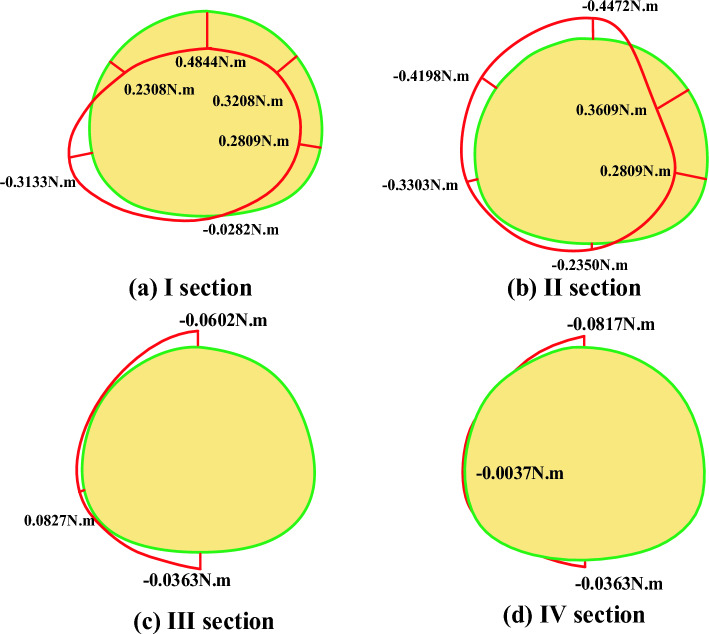
Moment increment after dislocation of fracture surface.

As shown in Fig. 17, the sections near the fault fracture surface (section I and section II) are obviously larger than those far away from the fault fracture surface, in which the maximum increment of the inner bending moment is 0.4844 N.m, which occurs at the vault of section I (footwall) and the maximum increment of the outer bending moment is 0.4472 N.m, which occurs at the vault of section II (footwall), far from the fault. From the comparison of the upper and lower plates, after the dislocation, the magnitude of the moment increment of the two sections is close, but the direction is different.

(3) safety factor

**Figure 18:**
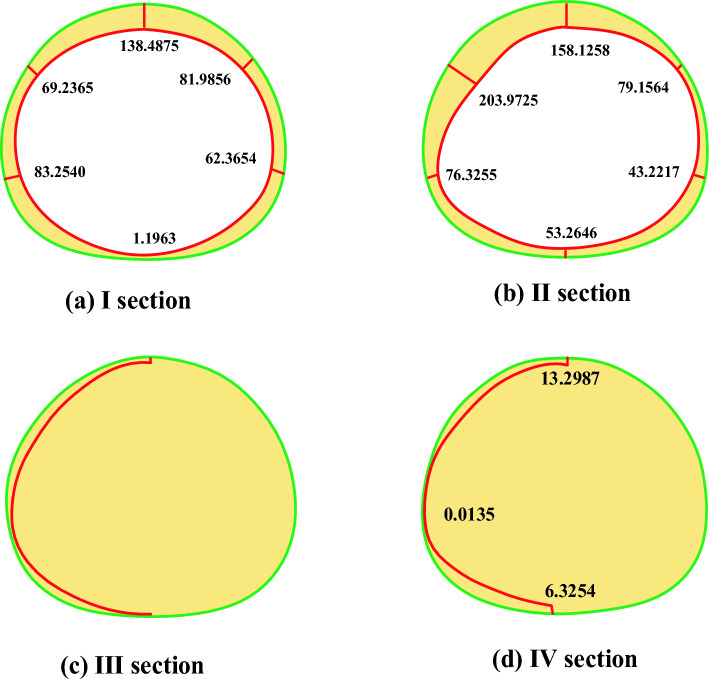
Reduction of safety factor after dislocation of fracture surface.

The change of safety factor reduction in different parts of each section before and after tunnel dislocation is shown in the Fig. 18. From the perspective of safety factor, the safety factor of the whole structure decreases sharply after dislocation, and the sections near the fault fracture surface (section I and section II) are obviously larger than those far away from the fault fracture surface, with the maximum decrease of 203, which occurs at the arch waist of section II (hanging wall). From the perspective of safety factor reduction values at each point, the tunnel is at the vault, arch waist and left and right arch feet. From the comparison between the upper and foot walls, the safety factor of the hanging wall is much lower than that of the foot wall after the dislocation occurs, and from the actual tunnel damage, the cracking of the hanging wall lining is more serious than that of the foot wall lining.

#### Longitudinal internal force

Data changes of lining longitudinal strain gauge before and after fault dislocation of static strain gauge are collected and converted into axial force and bending moment, as shown in Table 5.

**Table5:** Comparison of longitudinal internal forces of lining before and after fault dislocation without damping measures.

Section	Key position	Before and after dislocation Axial force difference (N)	Before and after dislocation Bending moment difference (Nm)
I	Vault	− 121.5844	− 0.1628
	Left arch waist	− 105.6938	− 0.0066
	Left arch foot	− 254.5031	0.0361
	Inverted arch	− 180.8156	0.1986
	Right arch waist	− 175.4156	0.2036
	Right arch foot	− 120.9938	0.1477
II	Vault	− 252.2531	− 0.1519
	Left arch waist	− 145.9125	− 0.0120
	Left arch foot	− 373.4719	− 0.1524
	Inverted arch	− 197.6625	0.0301
	Right arch waist	− 157.4156	− 0.1840
	Right arch foot	− 214.5938	− 0.0553
III	Vault	− 12.2625	− 0.0302
	Arch foot	− 36.7031	− 0.0603
	Inverted arch	− 17.6063	0.0008
IV	Vault	− 15.9313	− 0.0172
	Arch foot	4.2500	0.0039
	Inverted arch	− 39.4375	− 0.0281

**Figure 19:**
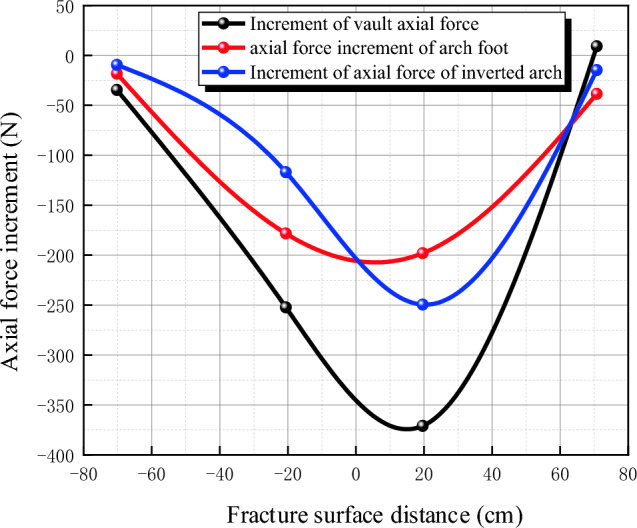
Relationship between axial force increment and fracture surface distance.

**Figure 20:**
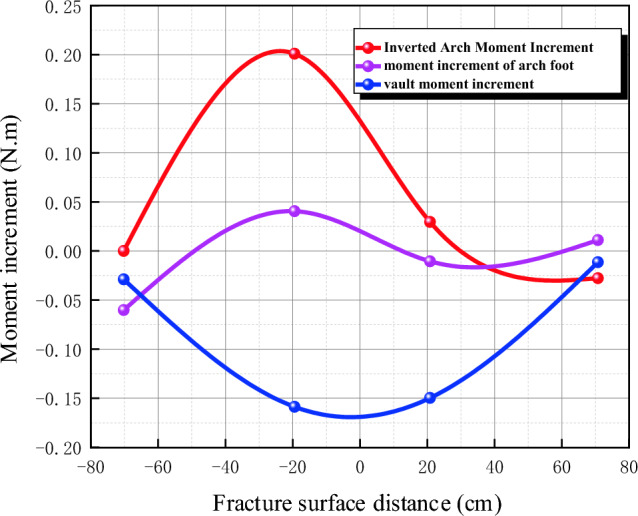
Relationship between Moment Increment and Fracture Surface Distance.

In order to reflect the relationship between the longitudinal internal force change and the location of the tunnel, the data are combined with the location of the fracture surface. The relationship between the axial force increment and the distance of the fracture surface is shown in Fig. 19, and the relationship between the bending moment increment and the distance of the fracture surface is shown in Fig. 20, where the abscissa is the fault fracture surface and the abscissa is the distance from the fracture surface. It can be seen from the figure that the increment of longitudinal axial force of the tunnel is basically divided by the fracture surface, and the closer it is to the fracture surface, the more obvious the increment is, in which the increment of longitudinal axial force at the left arch foot of the hanging wall reaches-373 N; At the same distance from the fault, the increment of the hanging wall is larger than that of the foot wall; The increment of longitudinal bending moment of tunnel is basically divided by the fracture surface, and the closer it is to the fracture surface, the more obvious it is. The increment of longitudinal bending moment at the inverted arch of footwall reaches 0.2 Nm, and the increment of footwall is larger than that of footwall at the same distance from the fault.

## Numerical simulation test

### Basic assumptions

(1) The surrounding rock is an isotropic continuous medium;

(2) Elastic model is selected as the constitutive model of lining concrete;

### Establishment of calculation model

**Figure 21:**
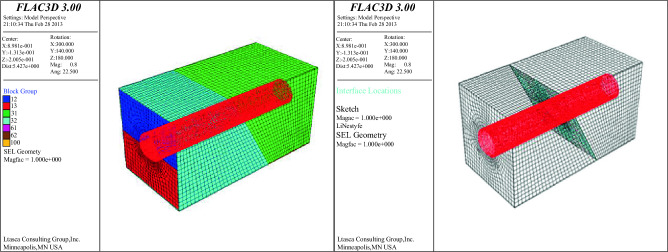
Relationship between calculation model and space.

The actual size of the model test is 2.0 m × 1.0 m × 1.0 m, and the relationship between the model and the space is shown in Fig. 21. The surrounding rock parameters of the tunnel are Grade IV, and the sections of the typical two-lane expressway tunnel and 250 km/h high-speed railway tunnel are used for lining. The model is built according to the similarity ratio of 1: 40, and the initial lining thickness is 1.5 cm. The elastic constitutive relation is adopted in this calculation, and the model has 51,150 elements and 54,366 nodes.

### Calculation parameters

Referring to the test parameters of gypsum, the shell element is used for simulation, and the whole model is wrapped by steel frame to simulate the boundary conditions. The elastic constitutive relation is adopted in this calculation, and the calculation parameters are selected according to Table 6.

**Table 6:** Calculation parameters.

name	Bulk density (kN/m^3^)	modulus of elasticity (Gpa)	Internal friction angle (°)	Cohesion (Mpa)	Poisson's ratio
Surrounding rock	17.5	0.1	–	–	0.30
liner	9.0	0.5	–	–	0.25
steel frame	78.5	30.0	–	–	0.25

For fault simulation, the interface element in flac3d is adopted, and the interface parameters are shown in Table 7.

**Table 7:** Contact surface parameters.

name	Normal stiffness (N/m)		Tangential stiffness (N/m)	Internal friction angle (°)	Cohesion (Mpa)	Tensile strength
interface	3.6 × 10^12^	3.6 × 10^12^		37	0.1	1000

Among them, according to the manual, the normal stiffness and tangential stiffness can be taken as 10 times of the equivalent stiffness of the "hardest" corresponding area around, that is:

$$k_{n} = k_{s} = 10\max \left[ {\frac{{K + \frac{4}{3}G}}{{\Delta z_{\min } }}} \right]$$

where K is the bulk modulus and G is the shear modulus, which $\Delta z_{\min }$ is the smallest dimension on the connecting area in the normal direction of the interface.

### Internal force response of lining structure

**Figure 22:**
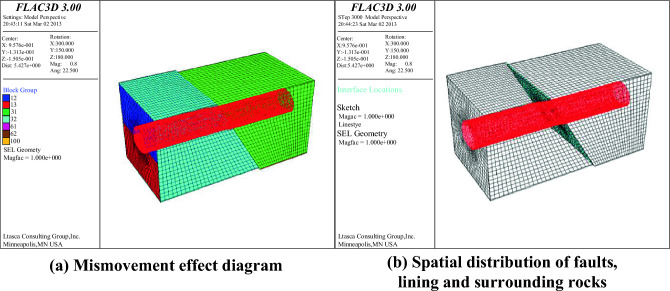
The upper and lower plates are staggered with each other.

The simulated effect is shown in Fig. 22. When forced displacement is applied to the upper and lower disks, the upper and lower disks begin to mismove each other, and the spatial location distribution of faults, linings and surrounding rocks begins to change .

**Figure 23:**
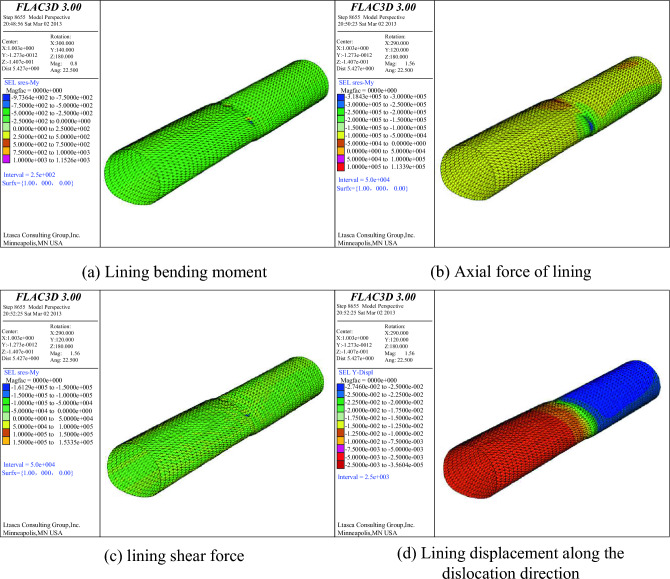
Internal Force and Displacement Diagram.

From the numerical simulation results, it is found that the lining bending moment, lining axial force, lining shear force and lining displacement along the dislocation direction all change, mainly concentrated in the active fault. The internal force and displacement of the whole tunnel lining structure are shown in Fig. 23.

### Comparison results of model test and numerical simulation

**Figure 24:**
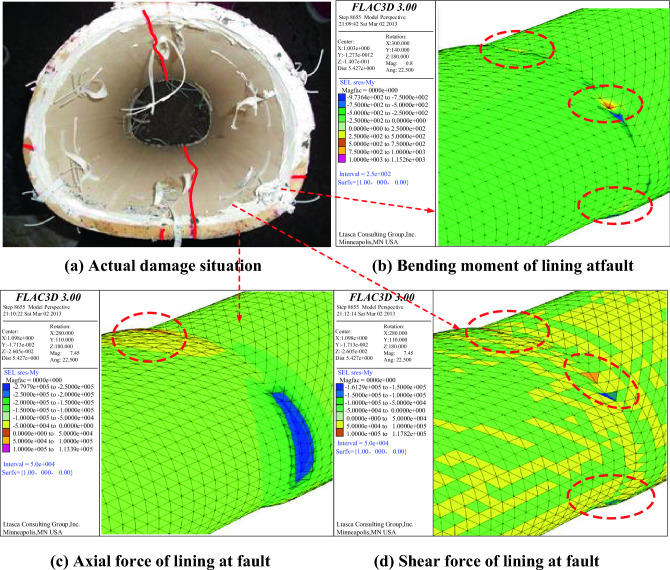
Comparison between model test and numerical simulation.

As shown in Fig. 24, the amplification of the internal force at the dislocation of the active fault can be seen from the internal force response diagram of the whole tunnel lining. The internal force changes of the lining caused by the dislocation of the fault are mainly concentrated near the active fault. From the enlarged display diagram of the internal force at the dislocation of the active fault, it can be seen that the internal force of the lining at the fault is concentrated at the vault, arch waist, arch foot and inverted arch. These characteristics are highly consistent with the failure characteristics of the model test lining.

## Comparative test under different conditions

### Test of tunnel not crossing faults

**Figure 25:**
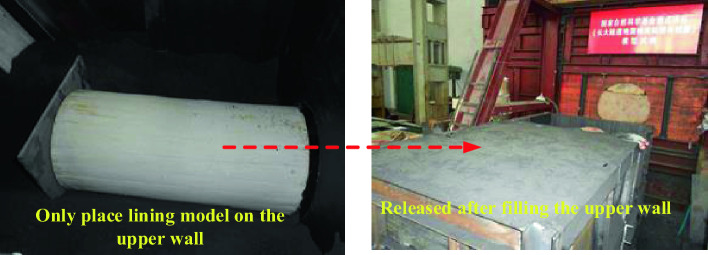
Comparative test of tunnel without crossing fault.

The failure characteristics of tunnel lining are generally longitudinal through cracks at the vault, arch foot and inverted arch. Through analysis, there may be two factors that have similar effects: (1) the shear and compression of the fracture surface when the fault moves; (2) The instantaneous reaction force caused by the fault hitting the bottom after dislocation. In order to analyze the main causes of the damage of lining structure, the experiment of designing a tunnel without crossing active faults is shown in Fig. 25, that is, the lining model is only placed in the hanging wall model box, and the length of the model can not reach the range of fault dislocation. When the fault is staggered, the fracture surface has little effect on the lining structure, and the structure mainly bears the instantaneous reaction force caused by the fault hitting the bottom.

**Figure 26:**
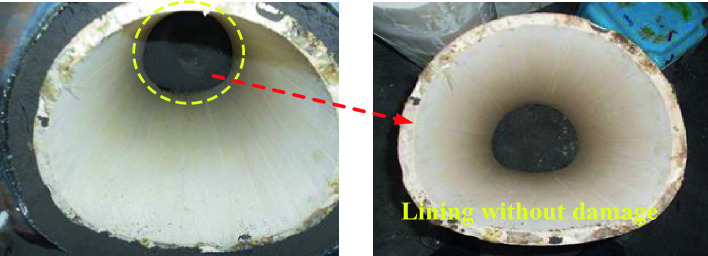
Lining without damage.

The damage of surrounding rock and lining structure after the test is shown in Fig. 26. After the hanging wall is staggered, the lining does not appear any cracks, and the structure is intact, which is completely different from the structural damage characteristics of the previous test. From the comparison of the two groups of tests, it is found that the main reason for the longitudinal through cracks in the basic test is the shearing and squeezing action of the fracture surface when the fault is dislocated, and the instantaneous reaction force generated after the fault touches the bottom has little effect, which is not enough to cause the lining to crack. However, if the lining has cracked under the action of the fault, the instantaneous reaction force may aggravate the damage of the tunnel lining.

### Fault test after reducing spring stiffness

**Figure 27:**
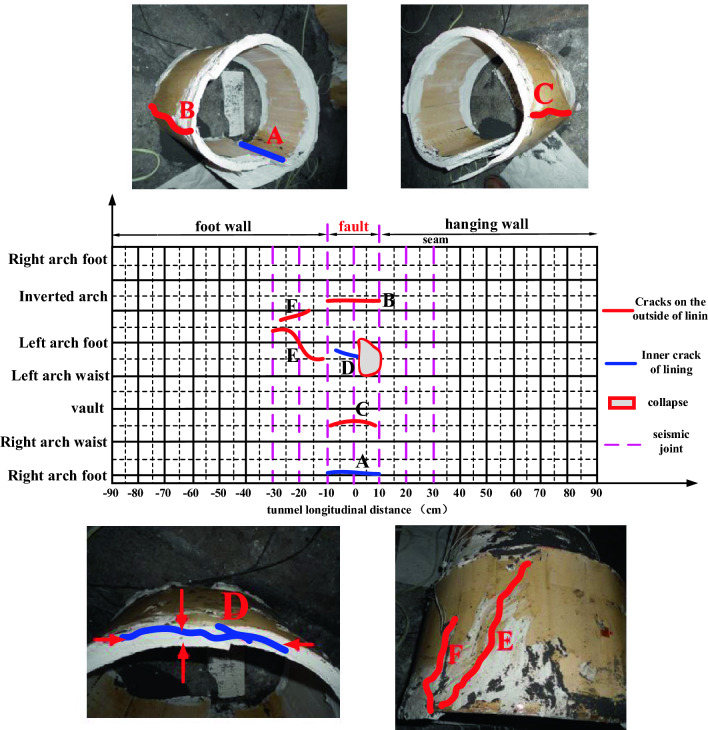
Crack Development and Failure.

The spring arranged under the upper plate can effectively simulate the repeated dislocation process of the upper and lower plates, and the stiffness of the spring directly affects the displacement and vibration time of repeated dislocation. In order to achieve the best test effect, reduce the spring stiffness and test the dislocation of the box with small stiffness. After the test, the damage of surrounding rock and lining structure is shown in the Fig. 27. From the failure form, after replacing the spring with small stiffness, the failure characteristics of the lining after fault dislocation are basically similar to those of the spring with large stiffness, and there are longitudinal through cracks at the inverted arch and arch foot, but the damage degree is slightly lighter than the former, that is to say, the spring becomes softer because of its stiffness, and its buffering ability to impact load is enhanced. At the same time, however, the test shows that the use of small stiffness spring will lead to the situation that the upper plate can not rebound effectively, and the mutual dislocation of the upper and lower plates is not obvious, with less reciprocating dislocation times, small displacement and short time, which can not achieve good test results.

### Fault test of lining model without reinforcement

**Figure 28:**
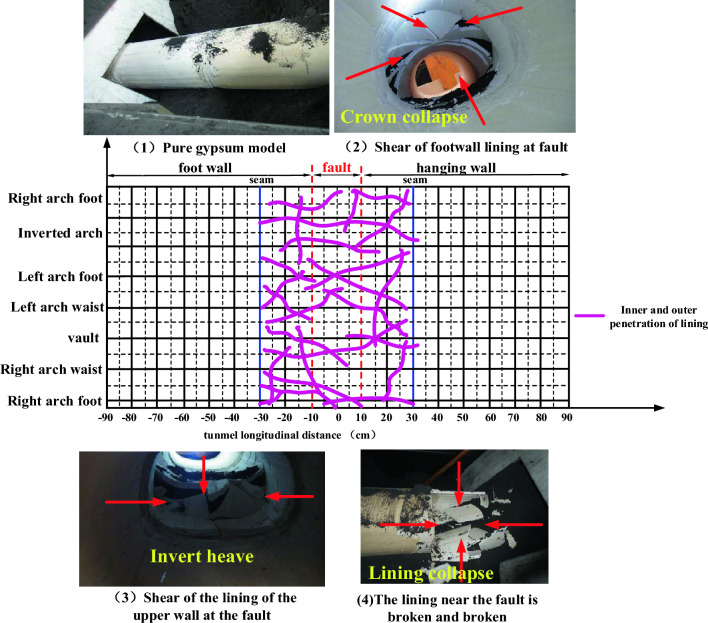
Crack Development and Failure.

In hard rock areas, many tunnels are lined with plain concrete, but if reinforced concrete is not used when passing through the fault fracture zone, it may cause very adverse effects. As shown in Fig. 28, pure gypsum is used to simulate the failure characteristics of plain concrete lining when passing through the fault fracture zone. Judging from the failure form, the pure gypsum lining has poor continuity because it has no reinforcement. After the fault moves, the lining near the fault is completely broken and the tunnel collapses. Compared with the previous reinforcement condition, the failure form has changed, showing complete collapse, large damage range and complete failure of the tunnel, which is the last result of the project. Therefore, brittle materials should not be used as lining for tunnels crossing active faults, but materials with good ductility and ability to buffer earthquake loads should be selected to ensure the collapse and maintainability of large earthquakes.

## Conclusion

In this paper, the failure characteristics of tunnel crossing active faults are studied by fault dislocation test, and indoor model tests are carried out by using the original fault sliding test box, as well as three groups of fault comparison tests: the tunnel does not cross faults, the spring stiffness is reduced and the model is not reinforced. The test device and test design can meet the basic requirements of simulating the mechanical behavior characteristics of tunnel crossing active faults, and the tunnel failure characteristics are obvious, which is basically consistent with the actual tunnel earthquake damage. The main conclusions are as follows:

1. In the test condition, after the hanging wall moves downwards, tree-like cracks occur on the surrounding rock surface of the hanging wall, with the maximum width of 2 cm and the depth of 8 cm, and the crack area is directly above the tunnel lining crossing the fault. Without damping joints, the lining is seriously damaged in the range of − 30–+ 30 cm (for the actual situation, − 12–+ 12 m) with the fault fracture surface as the center: through cracks appear in the inverted arch, and longitudinal through cracks appear in the left arch foot, right arch foot and vault. In addition, there are longitudinal through cracks in the lining inverted arch in the range of − 90 to − 30 cm in the footwall, and the fault dislocation has a great influence on the internal force of the tunnel.
2. From the point of view of axial force, all points show the characteristics of increasing compression, and the sections near the fault fracture surface (section I and section II) are obviously larger than those far away from the fault fracture surface, in which the maximum axial force compression increment is 198.8016 N, which occurs at the vault of section I(footwall). The whole tunnel shows circumferential compression at the fault position, and the axial force has no obvious change after being far away from the fault. From the comparison of the upper and lower plates, the axial force increment of the two sections is close after the dislocation occurs.
3. From the point of bending moment, the sections close to the fault fracture surface (section I and section II) are obviously larger than those far away from the fault fracture surface, in which the maximum increment of inner bending moment is 0.4844 N m, which occurs at the vault of section I(footwall) and the maximum increment of outer bending moment is 0.4472 N m, which occurs at the vault of section II (footwall), far from the fault. From the comparison of the upper and lower plates, after the dislocation, the magnitude of the moment increment of the two sections is close, but the direction is different.
4. From the perspective of safety factor, the safety factor of the whole structure decreases sharply after the dislocation, and the sections close to the fault fracture surface (section I and section II) are obviously larger than those far away from the fault fracture surface, with the maximum decrease of 203, which occurs at the arch waist of section II(the hanging wall). From the perspective of the safety factor reduction values of each point, the tunnel is more likely to crack at the vault, arch waist, left and right arch feet and inverted arch, It is in good agreement with the actual test structure. From the comparison between the upper and foot walls, the safety factor of the hanging wall is much lower than that of the foot wall after the dislocation occurs, and from the actual tunnel damage, the cracking of the hanging wall lining is more serious than that of the foot wall lining.

## Data Availability

All data, models, and code generated or used during the study appear in the submitted article.
